# “No Water, No Life. No Blue, No Green”

**DOI:** 10.3201/eid2404.AC2404

**Published:** 2018-04

**Authors:** Byron Breedlove, J. Todd Weber

**Affiliations:** Centers for Disease Control and Prevention, Atlanta, Georgia, USA

**Keywords:** about the cover, art science connection, art and medicine, emerging infectious diseases, Patricia Goslee, Water Prayer I, No Water, No Life. No Blue, No Green, antimicrobial resistance, biofilms, Vibrio cholerae, Legionella pneumophila, Pseudomonas aeruginosa, bacteria

**Figure:**
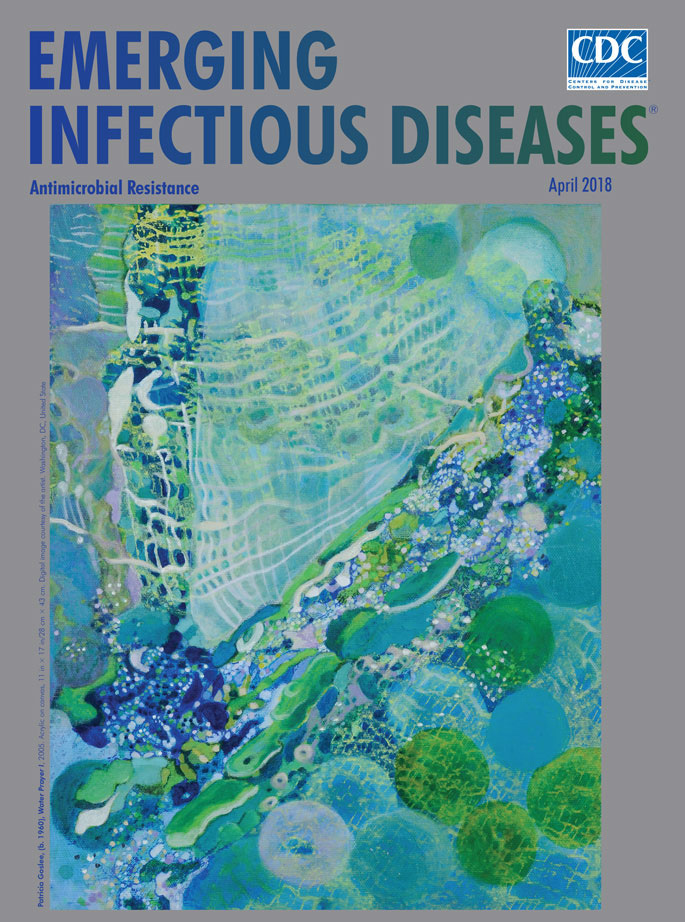
**Patricia Goslee, (b. 1960), Water Prayer I, 2005.** Acrylic on canvas, 11 in × 17 in/28 cm × 43 cm. Digital image courtesy of the artist.

“Thousands have lived without love, not one without water.”

—W. H. Auden, “First Things First”

Water is the most precious and essential natural resource. If unadulterated and at room temperature, it is tasteless, odorless (to humans), and transparent. Water sustains life, reshapes topography, provides passage and conveyance, and delineates and destroys geopolitical boundaries. Water comprises ≈71% of Earth's surface, and the United States Geological Survey estimates that Earth is covered by more than 332,500,000 cubic miles (mi^3^) of water. Archaeology, history, and anthropology corroborate that most civilizations originated near water. American marine biologist Sylvia Earl offers this perspective: “No water, no life. No blue, no green.”

Vivid blues and greens interspersed with layers of white splash across this month’s cover art, “Water Prayer I,” one of a series of water-related pieces from the portfolio of artist Patricia Goslee, who lives in Washington, DC, United States. Her abstract work points to the possibility of mutability and transformation in water. A hazy hatch work sweeps across the top of the painting and repeats in the lower right. Green and pale blue spheres of color float above the patterns. Dominating the image, a dense V-shaped amalgamation of speckled shapes—some uniform and others elongated—streaks diagonally across the center of the canvas while a column of undulating forms juts up along the left side.

Goslee’s water-themed painting can be viewed from divergent perspectives. It might be perceived as capturing a teeming collection of microorganisms inhabiting a drop of water. Conversely, it could suggests the proverbial 10,000-foot view, the stretched convergence of a city and river delta, interlaced with roads, canals, and lakes and dotted with buildings, fields, forests, and towns as viewed from an airplane window.

It may be a viewer’s choice, for Goslee, who favors a style that is colorful and intuitive, approaches her painting without a preconceived plan. In her words, “It’s a blank slate every time I start. I make marks, move and pour paint on the canvas. Sometimes I use spray paint, and sometimes I draw on the surface of the paintings. It all evolves and is ultimately a practice that allows me to process my experience of the world.” (P. Goslee, pers. comm., 2018 Feb 11.) 

 “Flow” was an exhibition of Goslee’s works displayed at the District of Columbia Arts Center in 2009–2010. Notes from this exhibition also offer perspective into her style of painting, “The most obvious unifying element in Goslee’s mixed media work is pattern: layers of color and form operate as a visual metaphor for layers of awareness. The results achieved often depict isolated moments, visualizations.”

Water is essential for life and for preserving health, but under certain circumstances, it can be the reservoir and conduit for pathogens that can lead to disease and death. Water is used in myriad ways to maintain hygiene, arguably handwashing being most critical for preventing the spread of organisms responsible for diseases as diverse as influenza and other respiratory infections, diarrheal disease, and healthcare-associated infections in hospitals. Water is critical for sterilization: steam under pressure has a long history of use for sterilization to prevent the spread of infections by reusable surgical instruments.

Water has been the source for infections of international and local importance. *Vibrio cholerae*, which preys on areas without adequate access to clean water and sanitation systems, has been responsible for 7 pandemics since the 19th century, killing millions of people across the globe. This organism remains endemic to many countries. *Legionella pneumophila* is transmitted by inhalation of contaminated aerosols from cooling towers, decorative fountains, hot water tanks, large plumbing systems, and the like. More recently, complex devices that use water and water drains have been identified as the site of biofilms harboring pathogens. Biofilms may form on a variety of water-associated surfaces, including living tissues, indwelling medical devices, industrial or potable water system piping, and natural aquatic systems. Surgical site infections caused by nontuberculous mycobacteria associated with the heater–cooler devices used during cardiac surgery have been reported internationally. *Pseudomonas aeruginosa* and other highly resistant organisms have been responsible for outbreaks associated with the waste and tap water systems in healthcare institutions.

Antibiotics themselves can contaminate water. A group of researchers discovered concentrations of pharmaceuticals, including levels of ciprofloxacin greater than those found in the blood of humans taking this antibiotic, in effluent from a water treatment plant that served around 90 drug manufacturers in India. They studied bacteria in river sediments and found genetic materials that could potentially confer resistance to ciprofloxacin and other antibiotics.

One of the most abundant and indispensable compounds, water courses through art, literature, history, and science. A multitude of different names exist for water, and cataloguing these would prove an arduous, complicated endeavor. Spending a few minutes reflecting on Goslee’s “Water Prayer I” enables us to move beyond words and simply appreciate how important water is to life and health.
